# The prevalence of unclosed transverse foramina in the cervical spine of a South African population sample

**DOI:** 10.1007/s00276-026-03834-w

**Published:** 2026-02-16

**Authors:** Shahed Nalla, Naa’ilah Noorbhai, Glen J. Paton

**Affiliations:** 1 Department of Human Anatomy and Physiology, Faculty of Health Sciences, University of Johannesburg, Johannesburg, South Africa; 2 Department of Chiropractic, Faculty of Health Sciences, University of Johannesburg, Johannesburg, South Africa

**Keywords:** Transverse foramen, Cervical, Vertebra, Unclosed foramen, Variation, South African

## Abstract

**Purpose:**

Unclosed transverse foramina (UTF) represent anatomical variations of the cervical vertebrae that may influence vertebral artery, vein or sympathetic nerves, particularly at the level of the atlas (C1). This study aimed to determine the prevalence and distribution of UTF in a South African skeletal population and to evaluate associations with age, biological sex, and population affinity.

**Methods:**

A macroscopic osteological assessment of cervical vertebrae from 800 individuals was performed using a South African skeletal repository. UTF were identified based on incomplete osseous closure of the transverse foramen. Prevalence was assessed at individual and vertebral levels, and demographic associations were analyzed statistically.

**Results:**

UTF were identified in 17.4% of individuals and 2.9% of total vertebrae. The highest prevalence occurred at C1, followed by C3 and C6. Unilateral UTF were more common than bilateral, with a right-sided predominance. UTF were observed more frequently in males, individuals younger than 50 years, and those of Black African population affinity, with the highest prevalence in the Sotho subgroup. A weak but significant association with younger age was noted at the C2 and C7 levels (*p* < 0.05).

**Conclusion:**

UTF are common anatomical variants in this South African population, with prevalence influenced by vertebral level and demographic factors. Recognition of UTF is important for accurate radiological interpretation and for surgical and manual procedures involving the cervical spine, where altered neurovascular anatomy may affect procedural planning and safety.

## Introduction

The cervical spine consists of seven vertebrae (C1–C7), with C1, C2, and C7 classified as atypical and C3–C6 considered typical in morphology [12]. A distinguishing feature of cervical vertebrae is the transverse foramen (TF), an osseous canal within the transverse process that transmits the vertebral artery, accompanying veins, and sympathetic nerve fibers [14]. The TF provides critical protection to these neurovascular structures and is formed through the fusion of the true transverse process with a vestigial costal element [2–5].

An unclosed transverse foramen (UTF) is an anatomical variant characterized by incomplete osseous enclosure of the TF, resulting in partial exposure of the vertebral artery. This variant is most commonly described at the atlas (C1), although it may occur at other cervical levels [20]. Incomplete TF formation may alter the anatomical course or stability of the vertebral artery, with potential implications for vascular compression, surgical exposure, and image interpretation [2–5]. Given the vertebral artery’s essential role in posterior cerebral circulation, such variations have been associated with vertebrobasilar artery insufficiency, which may present clinically with vertigo, dizziness, ataxia, and visual disturbance [3–10].

Previous studies have reported wide variation in UTF prevalence across populations, with most investigations focusing exclusively on the atlas and providing limited data on the remainder of the cervical spine [1, 16, 19, 20, 29]. Comprehensive assessments across all cervical levels remain uncommon, and data from African populations are particularly scarce. In South Africa, existing studies are few and restricted in sample size or population representation, limiting their applicability to clinical practice [18].

The present study aimed to determine the prevalence and distribution of UTF across the cervical vertebrae in a South African skeletal population. Associations with vertebral level, laterality, age, biological sex, and population affinity were evaluated.

## Materials and methods

### Study design and setting

A descriptive osteological study was conducted to investigate the prevalence and anatomical features of the UTF in the human cervical spine. The investigation was performed on the modern skeletal material from the Raymond A. Dart Collection of Modern Human Skeletons (known as the Dart Collection), University of the Witwatersrand, Johannesburg, South Africa. The South African populations from which the Dart Collection is derived are extremely diverse in terms of ancestry, culture, linguistics, biology, and genetics [5, 23].

### Specimen selection

A total of 800 skeletons (of the available Dart sample of 1118 skeletons) were pre-selected based on the redefined inclusion criteria that ensured the selection of cervical vertebrae that met specific demographic conditions. All demographic information of the skeletons used in this study was based on the information contained in the researcher catalogue shared by the curator of the Dart Collection.

The sample was drawn from individuals with documented South African ancestry, categorized as either Black South African (BSA) or White South African (WSA). The BSA group comprised three ancestry subgroups (Zulu, Xhosa, and Sotho), while the WSA group was not further subdivided. Inclusion required individuals to be between 20 and 70 years of age at death, with a recorded biological sex of male or female, and a complete, articulable, and morphologically intact cervical column (C1–C7). Vertebrae exhibiting significant pathology (e.g., osteoarthritis, infectious lesions), ante-mortem trauma, fractures, congenital anomalies (e.g., block vertebrae), or post-mortem damage that compromised the transverse foramen were excluded. The final study sample consisted of 800 skeletons (460 male, 340 female) (Table 1).

**Table 1:** The prevalence of UTF (C1–C7) in the literature

Study	Country	No of cases	Total UTF present	C1 (%)	C2 (%)	C3 (%)	C4 (%)	C5 (%)	C6 (%)	C7 (%)
Varaglia [27]	Italy			14.5	8	2	0	2	6	0
Macalister [14, 15]	England			3.6	2.6	–	–	–	–	–
Le Double [12]	France	772	107	14	2.2	1.5	2.5	3.5	6	2
Hasebe [9]		100	6	6						
Dubreuil-Chambardel [8]	France	430	52	12.1	–	–	–	–	–	–
Bergman [2]		142	17	12						
De Sousa, Rodriguez and Dos Santos Ferreira [7]		200	16	8	–	–	–	–	–	–
Wysocki et al. [29]	Poland			9.4	1	–	–	–	–	–
Le Minor and Trost [13]	France			4.6	–	–	–	–	–	–
Billmann and Le Minor [3]	France	500	51	10.2	–	–	–	–	–	–
Travan et al. [26]	Italy	923		8.4	3.4	1.1	2	0	1.4	1.6
Karau and Odula [10]	Kenya	102	8	7.8						
Sultana et al. [24]	India	100	5	5						
Sanchis-Gimeno et al. [19]	South Africa	218	17	7.8						

### Morphological assessment

All seven cervical vertebrae from each person were assessed macroscopically under standard laboratory lighting. Vertebrae were arranged in anatomical sequence from C1 to C7, and the TF at each level were examined. The TF was classified as closed, defined by a complete bony ring, or unclosed (UTF), characterized by incomplete ossification presenting as a bony groove. Where present, UTF was recorded as unilateral, specifying left or right, or bilateral, and documented according to vertebral level. The demographics of the total sample are summarized in Table 2.

**Table 2:** Frequencies and percentages of the total sample

Demographic component	Frequency (*n*)	Percentage (%)
Age		
Younger than 50 years	404	50.5
50 years and older	396	49.5
Total Age	800	100
*Biological sex*		
Male	460	57.5
Female	340	42.5
Total sex	800	100
*Population affinity*		
Black	600	75
White (“European”)	200	25
Total population affinity	800	100
*Ancestry*		
Zulu	244	30.5
Xhosa	156	19.5
Sotho	200	25
White (“European”)	200	25
Total ancestry	800	100

#### Data management and reliability

All observations were recorded in a standardized digital protocol. Both intra-observer and inter-observer reliability was assessed by re-examining a random subset of 80 vertebrae (10% of sample) after a two-week interval. Inter-observer reliability was evaluated using a second anatomist who independently assessed the same subset as the principal researcher. Agreement was quantified using Cohen’s kappa (κ) statistic. For intra-observer reliability was 1.00 (100%), which indicates perfect agreement. Inter-observer reliability was 0.98 (98%), which indicates almost perfect agreement.

## Statistical analysis

Descriptive statistics expressed the prevalence of UTF as counts and percentages per vertebral level, side, sex, and population group. Associations between categorical variables (e.g., sex and UTF presence) were analyzed using the Chi-square test or Fisher’s exact test, as appropriate. A* p*-value < 0.05 was considered statistically significant. Analyses were performed using SPSS Statistics version 28 (IBM Corp., Armonk, NY, USA).

## Results

### Prevalence of unclosed transverse foramina

**Fig. 1:**
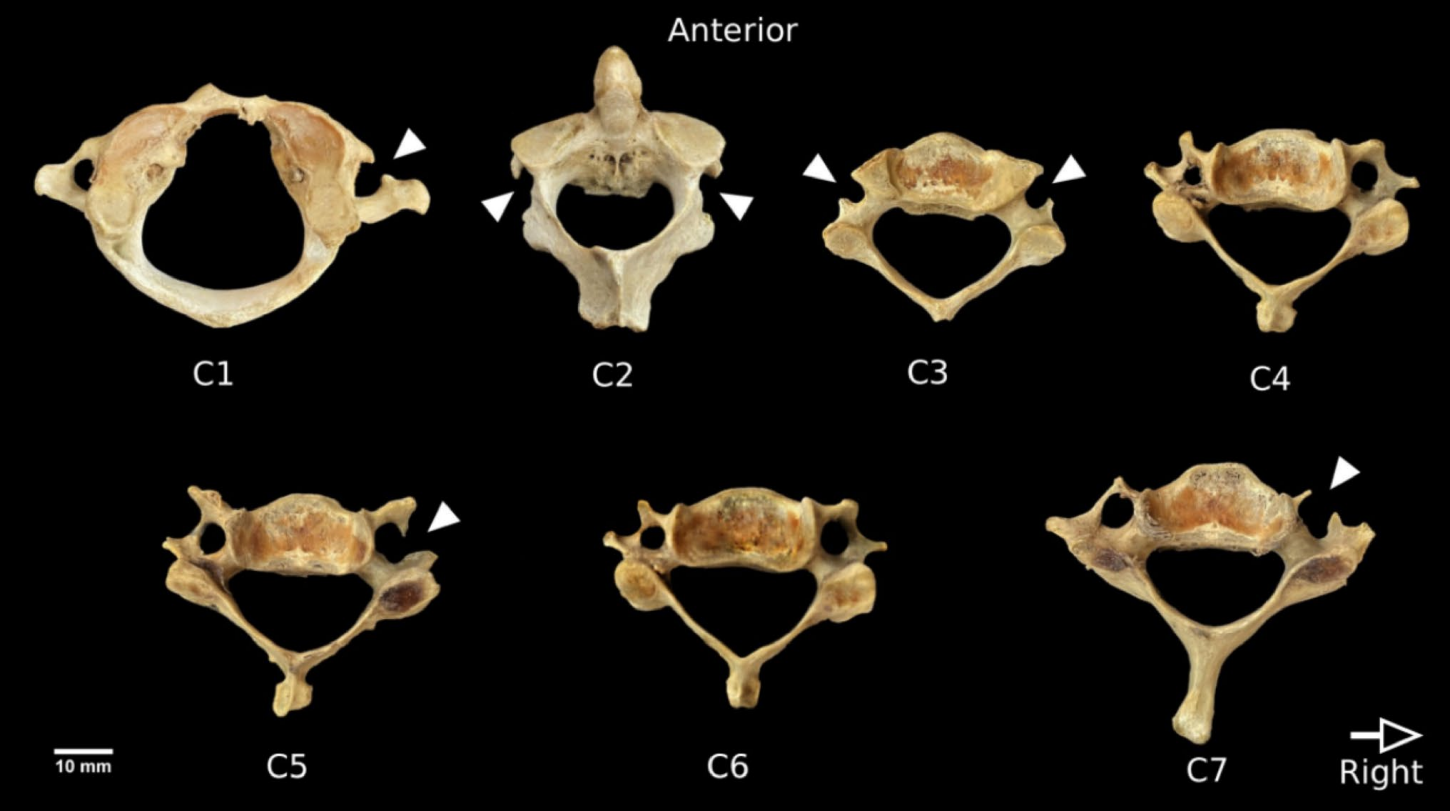
A superior view of all cervical vertebrae of a 22-year-old male. Five cervical vertebrae contained unclosed transverse foramina (UTF). Arrowheads indicate the side of the UTF

Of the 800 individuals examined, 139 (17.4%) exhibited at least one unclosed transverse foramen (UTF). Most affected individuals presented a single UTF (86.3%, *n* = 120). Cases with two (*n* = 16), three (*n* = 2), and five (*n* = 1) UTF were less frequent (Fig. 1).

At the vertebral level, 163 of the 5600 cervical vertebrae examined exhibited a UTF, yielding an overall prevalence of 2.9%. The atlas (C1) demonstrated the highest prevalence, accounting for 42.3% (69/163) of all UTF identified at the vertebral level. The lowest prevalence was observed at C5, where UTF occurred in only 9 individuals (1.1%). Unilateral UTF was more frequent than bilateral involvement. Detailed frequencies for unilateral and bilateral presentations are provided in Table 3.

**Table 3:** The number of individual skeletons containing at least one UTF

Number of UTF present per individual	Number of individuals (*n*/800)
1	120 (15%)
2	16 (2%)
3	2 (0.3%)
4	0
5	1 (0.1%)
6	0
7	0
Total	139 (17.4%)

### Prevalence by demographic factors

#### Biological sex

Within the sample of 460 males and 340 females, UTF occurred in 102 males (62.6% of all UTF) and 61 females (37.4%). At C1, UTF prevalence was 9.8% in males and 7.1% in females (Table 4).

**Table 4:** The prevalence of UTF in the cervical vertebra

Vertebra	Unilateral (Left)	Unilateral (Right)	Bilateral	Total (*n*/800)	Total (*n*/163)
C1	19 (2.4%)	29 (3.6%)	21 (2.6%)	69 (8.6%)	69 (42.3%)
C2	3 (0.4%)	8 (1%)	6 (0.8%)	17 (2.1%)	17 (10.4%)
C3	9 (1.1%)	7 (0.9%)	4 (0.5%)	20 (2.5%)	20 (12.3%)
C4	6 (0.8%)	2 (0.3%)	2 (0.3%)	10 (1.3%)	10 (6.1%)
C5	5 (0.6%)	2 (0.3%)	2 (0.3%)	9 (1.1%)	9 (5.5%)
C6	7 (0.9%)	9 (1.1%)	3 (0.4%)	19 (2.4%)	19 (11.7%)
C7	7 (0.9%)	11 (1.4%)	1 (0.1%)	19 (2.4%)	19 (11.7%)
Total (n/5600)	56 (1%)	68 (1.2%)	39 (0.7%)	163 (2.9%)	163

#### Age

UTF was observed in 94 individuals younger than 50 years (57.7% of all UTF) and 69 individuals aged 50 years or older (42.3%). At C1, prevalence was 9.4% in the younger group and 7.8% in the older group.

#### Population affinity and ancestry

**Fig. 2:**
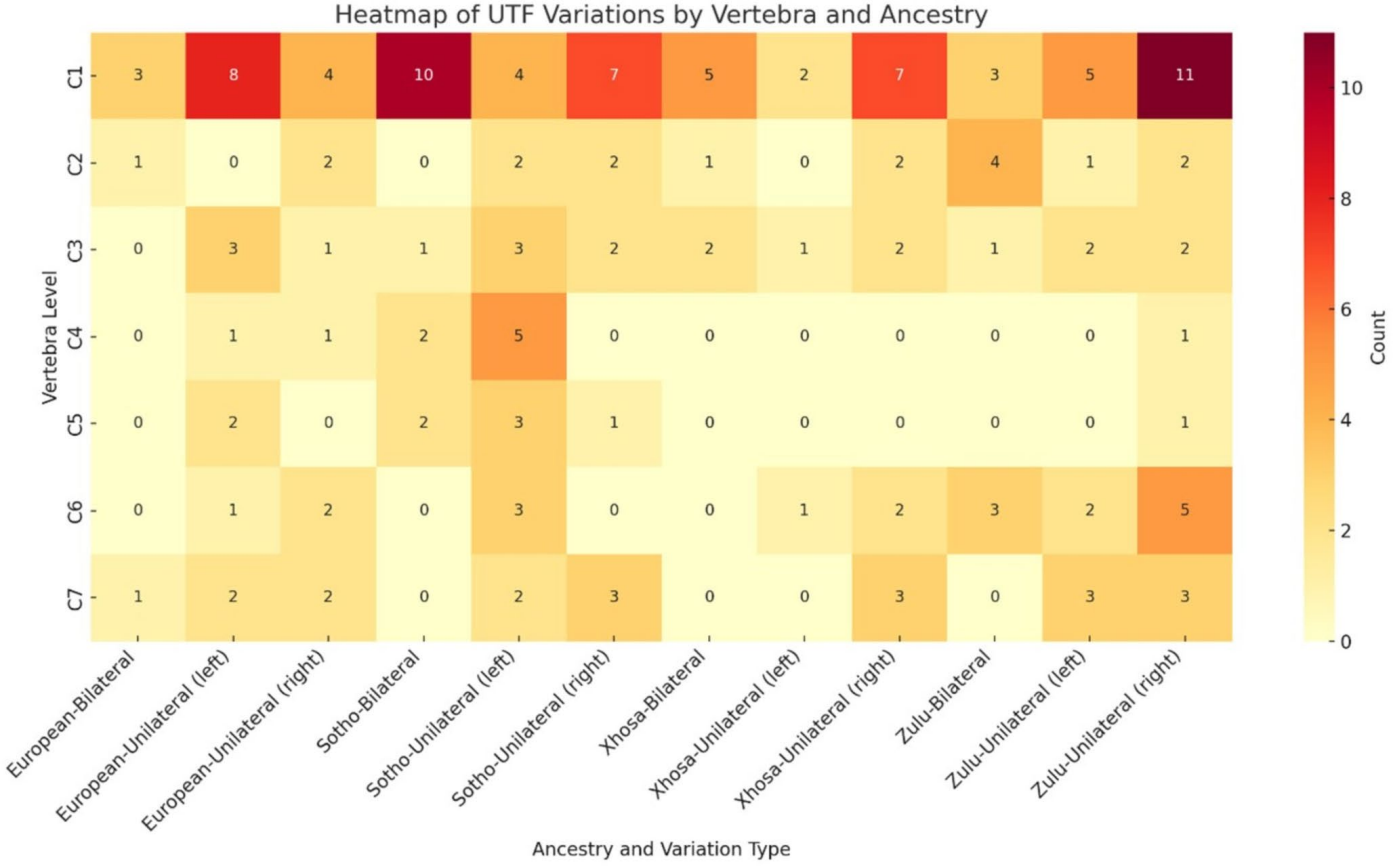
Heatmap of unclosed transverse foramina by spinal level and ancestry

Within the cohort of 600 Black South African (BSA) and 200 White South African (WSA) individuals, 79.1% of UTF cases occurred in the BSA group. At C1, UTF prevalence was 9.0% in the BSA group and 7.5% in the WSA group. UTF counts among BSA ancestry subgroups were highest for Sotho individuals (*n* = 52), followed by Zulu (*n* = 49) and Xhosa (*n* = 28). Across all groups, unilateral UTF was more common than bilateral presentation (Fig. 2).

#### Statistical analyses

No statistically significant associations were identified between UTF presence and biological sex, population affinity, or ancestry at any cervical level. Descriptive trends showed UTF occurred most frequently in individuals of Sotho ancestry at C1, C3, C4, and C5, and in the Zulu group at C2, C6, and C7.

A statistically significant but weak association was identified between age and UTF presence at C2 (*p* = 0.047) and C7 (*p* = 0.033; Cramér’s V = 0.072). Notably, no UTF was observed at C4 or C5 in individuals of Xhosa ancestry. All statistical results are summarized in Table 5.

**Table 5:** Values of each vertebra (C1–C7) split by demographics

Vertebra number	Age (*P*-value)	Sex (*P*-value)	Population affinity (*P*-value)	Ancestry (χ^2^, df, *P*-value)
C1	0.45	0.20	0.56	χ^2^ = 1.455 df = 3, *p* = 0.70
C2	0.05*	0.33	0.58	χ^2^ = 1.070, df = 3, *p* = 0.78
C3	0.50	0.50	0.80	χ^2^ = 0.932 df = 3, *p* = 0.82
C4	1	0.20	1	χ^2^ = 11.674, df = 3, *p* = 0.01
C5	1	1	1	(χ^2^ = 9.246 df = 3, *p* = 0.03
C6	0.25	0.82	0.43	χ^2^ = 4.584 df = 3, *p* = 0.21
C7	0.03*	0.65	1	χ^2 ^= 0.172 df = 3, *p* = 0.98

Significant* P*-values are indicated in bold with an asterisk

## Discussion

The prevalence of UTF in the cervical spine has been reported as low, ranging between 3.6% and 15.2% in the literature [5, 16, 19, 29]. This informed the decision to utilise a large sample size in the present study. The prevalence rate of UTF in this study was 17.4% at the individual level (139/800) and 2.9% at the vertebral level (163/5600). When considering only the atlas, the prevalence was 8.6% (69/800). Variability in reported prevalence rates may be attributed to differences in study populations, sample sizes, methodologies, and genetic, environmental, or geographic factors influencing skeletal development.

In total, this study examined 5600 cervical vertebrae from 800 cervical spines. By contrast, earlier studies examined substantially smaller samples, typically between 100 and 500 vertebrae [2, 20, 18; Table 1]. Accordingly, the larger sample size in this study enhances the statistical power and robustness of the findings.

### Demographics

This study provides a comprehensive anatomical analysis of the unclosed transverse foramen (UTF), a variant of direct clinical significance. The principal findings confirm its status as a frequent anatomical variant with a distinct distribution.

The recording of population affinity is an important demographic aspect of any skeletal collection. In the Southern African region, population migrations during its history have involved populations throughout the African continent as well as from Europe and Asia [17, 21]. Thus, the South African populations from which the Dart Collection is derived are extremely diverse in terms of ancestry, culture, linguistics, biology, and genetics. The following population affinity groups were used in the study: Black South African (Zulu, Xhosa, Sotho) and WSA (“European”). These specific BSA groups were chosen because they are part of the three largest South African ethnic groups [5, 6, 23]. Population affinity refers to an estimation of group membership that indicates how morphologically or genetically similar an individual is to a well-defined group [22]. This is determined based on a measure of statistical distance. Ancestry refers to one’s ancestral origins [11].

Male skeletons predominate in documented skeletal collections both in South Africa and internationally. In the Raymond A. Dart Collection, males comprise 71% of individuals, while females account for 29% [5]. A similar imbalance is observed in the Pretoria Bone Collection (75.5% male) [11] and the University of Tennessee Donated Skeletal Collection (64% male) [4]. This consistent pattern has been attributed to historical and socioeconomic factors, particularly labor migration and higher rates of unclaimed male remains, whereas females are more likely to maintain family ties that facilitate post-mortem repatriation [5, 11, 18].

### Principal findings and anatomical patterns

The central finding of this study is the identification of the UTF as a common anatomical variant. The atlas (C1) was the most commonly affected vertebra (prevalence: 8.6%), consistent with existing literature [1, 16]. However, the descending order of prevalence in our sample (C1 > C3 > C6 ≈ C7 > C2 > C4 > C5) differs from patterns reported in other populations [16], suggesting potential population-specific tendencies. Furthermore, unilateral presentations were more common than bilateral, with a slight, non-significant predominance on the right side, contrasting with some prior studies [20]. See Table 6 for comparison and significant findings for comparison of UTF.

**Table 6:** UTF comparison in this study and literature

Study	Country	Vertebral number						
		C1 (%)	C2 (%)	C3 (%)	C4 (%)	C5 (%)	C6	C7 (%)
This study	South Africa	8,6	2,1	2,5	1,3	**1**,**1%**	**2**,**4**	2,4
Travan et al. [14]	Italy	8,4	3,4	1,1	2	**0**	**1**,**4%**	1,6
Le Double [10]	France	14	2,2	1,5	2,5	**3**,**5**	**6%**	2

Significant values are bolded

Our overall vertebral-level prevalence of 2.9% falls within the lower end of the reported spectrum (3.6%–15.2%) [2, 12–14, 25], a variability underscoring the influence of sample characteristics. The strength of this study is its large, well-defined sample of 5600 vertebrae, providing greater statistical power than many prior studies [3, 10, 19]. We found statistically significant association between UTF and age for C2 and C7 vertebral levels, but no correlations exist with biological sex. The literature reports that UTF is a developmental trait established by the completion of ossification [24–26].

### Clinical and surgical implications: a call for vigilance

The central finding of this study is the identification of the UTF as a common anatomical variant, with a prevalence and distribution that both align with and diverge from key international reports. Consistent with studies across diverse populations, the atlas (C1) was the most commonly affected vertebra in our sample, with a prevalence of 8.6% [1].

However, the descending order of prevalence in our South African cohort (C1 > C3 > C6 ≈ C7 > C2 > C4 > C5) reveals a distinct anatomical pattern. This contrasts markedly with the sequence reported in a large Italian archaeological sample (C1 > C2 > C4 > C7 > C6 > C3 > C5) [1] and a classic French study (C1 > C6 > C5 > C4 > C7 > C2 > C3) [16]. Notably, our data show a relatively higher prevalence at C3 and C7, and a lower prevalence at C2 and C5, compared to these European samples (See Table 5). This divergence suggests potential population-specific morphological tendencies in the development of the cervical transverse process.

Although vertebral artery caliber and patency were not assessed, the frequent occurrence of UTF raises clinically relevant questions regarding associated vascular variation. As the transverse foramen serves as the osseous conduit for the vertebral artery, morphological deviations may coexist with arterial hypoplasia, asymmetry, or anomalous course. Valenzuela-Fuenzalida et al. demonstrated that unilateral vertebral artery hypoplasia is substantially more common than bilateral involvement and often exhibits side dominance [28], paralleling the predominance of unilateral UTF and the subtle right-sided tendency observed in this study.

From a surgical and radiological perspective, the coexistence of bony and vascular variants may increase procedural risk. An unclosed transverse foramen, particularly at upper cervical levels, may alter the expected bone–artery relationship during cervical instrumentation, thereby increasing the risk of iatrogenic vascular injury. Reduced collateral circulation associated with vertebral artery hypoplasia may further magnify the clinical consequences of arterial compromise [27].

Furthermore, unilateral presentations were more common than bilateral in our study, with a slight, non-significant predominance on the right side, a finding that also contrasts with some prior reports [20]. We found no statistically significant association between UTF prevalence and age or biological sex, aligning with the consensus that it is a developmental trait established by the completion of ossification [18, 23].

## Limitations

This study has limitations inherent to its design. As an osteological study, it describes bony morphology but cannot correlate findings with the in vivo course, caliber, or patency of the vertebral neurovascular structures or adjacent soft tissues. Additionally, the absence of radiological correlation was not possible. As noted, this study is limited to dry bones, which precludes direct assessment of vertebral artery hypoplasia. Future investigations could compare the geographic and ethnic distribution of vertebral artery hypoplasia with the bony variations observed, such as unclosed transverse foramina, to explore potential correlations between skeletal and vascular anatomy. The demographic composition of the skeletal collection, while large and well-documented, may not be fully representative of all global populations.

## Future implications

Future research should focus on bridging anatomical knowledge with clinical practice. Priority directions include radiologic-Clinical Correlation Studies: Prospective studies using pre-operative CT to document UTF prevalence in surgical cohorts and correlate findings with intraoperative anatomy and surgical outcomes. Improved Pre-Operative Imaging Protocols: Development and validation of standardized imaging protocols or checklists to ensure consistent reporting of UTF and other vascular foramen variants. Investigation of Aetiology: Research into the genetic and embryological foundations of UTF to better understand its development and potential associations with other anatomical variations.

## Conclusion

This study assessed the prevalence of unclosed transverse foramina (UTF) in the cervical spine within a South African population, examining 800 skeletons and 5600 cervical vertebrae. The findings revealed that UTF was most common in C1 (8.6%) and least prevalent in C5 (1.1%), with unilateral occurrences more frequent than bilateral. UTF was more prevalent in younger individuals and males, with the Black South African population showing the highest prevalence. This research is the first of its kind in South Africa and provides essential insights into the anatomical variation of UTF. These findings have important implications for healthcare practitioners, particularly chiropractors, radiologists, and surgeons. Knowledge of UTF is relevant for radiological interpretation, surgical planning, and interventions in the cervical spine, as variations may affect the course of the vertebral artery or alter landmarks used in imaging and surgery. While UTF can be associated with vertebrobasilar insufficiency, there is no evidence of increased risk of VBAI stroke due to cervical manipulation. Understanding these variations can improve patient safety, enhance diagnostic accuracy, and guide clinical and surgical decision-making.

## Data Availability

Availability of data associated with the manuscript is available on request.

## References

[1] Aziz J, Morgan M (2018) Morphological study of the foramen transversarium of the atlas vertebra among the Egyptian population and its clinical significance. Int J Anat Res 6:5219–5224. 10.19080/APBIJ.2018.04.555642

[2] Bergman P (1967) On varieties of the atlas in man. Folia Morphol 26:129–139 5299695

[3] Billmann F, Le Minor JM (2009) Transverse foramen of the atlas (C1) anteriorly unclosed: a misknown human variant and its evolutionary significance. Spine 34:E422–E426. 10.1097/BRS.0b013e3181a2e07e 19454993 10.1097/BRS.0b013e3181a2e07e

[4] Campanacho V, Alves Cardoso F, Ubelaker DH (2021) Documented skeletal collections and their importance in forensic anthropology in the United States. Forensic Sci 1:228–239. 10.3390/forensicsci1030021

[5] Dayal MR, Kegley AD, Štrkalj G, Bidmos MA, Kuykendall KL (2009) The history and composition of the Raymond A. dart collection of human skeletons at the university of the Witwatersrand, Johannesburg, South Africa. Am J Phys Anthropol 140:324–335. 10.1002/ajpa.21072 19382178 10.1002/ajpa.21072

[6] De Gama BZ, Jones DG, Bhengu TT, Satyapal KS (2020) Cultural practices of the Zulu ethnic group on the body and their influence on body donation. Anat Sci Educ 13:721–731. 10.1002/ase.1930 32077216 10.1002/ase.1950

[7] De Sousa CA, Rodriguez M, Dos Santos Ferreira A (1989) Contribuição para o estudo da charneira occipito-vertebral. Arquivos De Anat E Antropologia 40:209–226

[8] Dubreuil-Chambardel L (1921) L’Atlas. Vigot, Paris

[9] Hasebe K (1913) Die Wirbelsäule der Japaner. Z für Morphologie Und Anthropologie 15:259–380

[10] Karau PB, Odula P (2013) Some anatomical and morphometric observations in the transverse foramina of the atlas among Kenyans. Anat J Afr 2:61–66

[11] L’Abbé EN, Krüger GC, Theye CEG, Hagg AC, Sapo O (2021) The Pretoria bone collection: a 21st century skeletal collection in South Africa. Forensic Sci 1:220–227. 10.3390/forensicsci1010020

[12] Le Double AF (1912) Traité des variations de la colonne vertébrale de l’homme. Paris, Vigot

[13] Le Minor JM, Trost O (2004) Bony Ponticles of the atlas (C1) over the groove for the vertebral artery in humans and primates: polymorphism and evolutionary trends. Am J Phys Anthropol 125:16–29. 10.1002/ajpa.10270 15293328 10.1002/ajpa.10270

[14] Macalister A (1893) Notes on the development and variations of the atlas. J Anat Physiol 27:519–542 PMID: 17232056 17232056 PMC1328304

[15] Macalister A (1894) The development and variations of the second cervical vertebrae. J Anat Physiol 28:257–268 PMID: 17232080 17232080 PMC1328386

[16] Murlimanju BV, Prabhu LV, Shilpa K, Rai R, Dhananjaya KV, Jiji PJ (2011) The prevalence, embryological basis, morphology, and surgical significance of cervical spine accessory transverse foramina. Turk Neurosurg 21:384–387. 10.5137/1019-5149.JTN.4047-10.0 21845576 10.5137/1019-5149.JTN.4047-10.0

[17] Nurse GT, Weiner JS, Jenkins T (1985) The peoples of Southern Africa and their affinities. Clarendon, Oxford

[18] Paton GJ, Williams SA, Nalla S, Louw GJ (2024) Lumbosacral transitional vertebrae of 3096 individuals: prevalence and morphology in a South African population and its association with population affinity. Transl Res Anat 35:100281. 10.1016/j.tria.2024.100281

[19] Sanchis-Gimeno JA, Llido S, Perez-Bermejo M, Nalla S (2018) Prevalence of anatomic variations of the atlas vertebra. Spine J 18:2102–2111. 10.1016/j.spinee.2018.06.022 29960109 10.1016/j.spinee.2018.06.352

[20] Scheuer L, Black S (2000) Developmental juvenile osteology. Academic, San Diego

[21] Soodyal H (2006) The prehistory of africa: tracing the lineage of modern man. Johannesburg, Jonathan Ball Publishers

[22] Spradley MK, Jantz RL (2021) What are we really estimating in forensic anthropological practice, population affinity or ancestry? J Forensic Anthropol 4:309–318. 10.5744/fa.2021.0017

[23] Statistics South Africa (2023) Media release: Census 2022 population count results. Pretoria, Statistics South Africa. Accessed 3 Sept 2025

[24] Sultana Q, Avadhani R, Varalakshmi KL, Shariff MH (2015) Variations of foramen transversarium in atlas vertebrae: a morphological study with its clinical significance. J Health Allied Sci NU 5:80–83. 10.1055/s-0040-1709822

[25] Taitz C, Nathan H, Arensburg B (1978) Anatomical observations of the foramina transversaria. J Neurol Neurosurg Psychiatry 41:170–176. 10.1136/jnnp.41.2.170 632823 10.1136/jnnp.41.2.170 PMC492985

[26] Travan L, Saccheri P, Gregoraci G, Mardegan C, Crivellato E (2015) Normal anatomy and anatomic variants of vascular foramens in the cervical vertebrae: a paleo-osteological study and review of the literature. Anat Sci Int 90:308–323. 10.1007/s12565-014-0270-x 25576169 10.1007/s12565-014-0270-x

[27] Varaglia S (1885) Di alcune varietà ossee del tronco. Giornale Della Reale Accad Di Med Di Torino 8:1–9

[28] Valenzuela-Fuenzalida JJ, Rojas-Navia CP, Quirós-Clavero AP et al (2024) Anatomy of vertebral artery hypoplasia and its relationship with clinical implications: a systematic review and meta-analysis of prevalence. Surg Radiol Anat 46:963–975. 10.1007/s00276-024-03367-5 38762843 10.1007/s00276-024-03377-y

[29] Wysocki J, Bubrowski M, Reymond J, Kwiatkowski J (2003) Anatomical variants of the cervical vertebrae and the first thoracic vertebra in man. Folia Morphol 62:357–363 PMID: 14655117 14655117

[30] Zibis A, Mitrousias V, Galanakis N, Chalampalaki N, Arvanitis D, Karantanas A (2018) Variations of transverse foramina in cervical vertebrae: What happens to the vertebral artery? Eur Spine J 27:1278–1285. 10.1007/s00586-018-5523-2 29455293 10.1007/s00586-018-5523-2

[31] Zibis AH, Mitrousias V, Baxevanidou K, Hantes M, Karachalios T, Arvanitis D (2016) Anatomical variations of the foramen transversarium in cervical vertebrae: findings, review of the literature, and clinical significance during cervical spine surgery. Eur Spine J 25:4132–4139. 10.1007/s00586-016-4738-3 27554348 10.1007/s00586-016-4738-3

